# Manuscript Rejection: Causes and Remedies

**DOI:** 10.4103/0975-1483.62205

**Published:** 2010

**Authors:** Javed Ali

**Affiliations:** *Department of Pharmaceutics, Faculty of Pharmacy, Jamia Hamdard, Hamdard Nagar, New Delhi – 110 062, India E-mail: javedaali@yahoo.com*

Research studies performed in the field of pharmaceutical sciences are often attempted to be converted into published manuscripts. A research manuscript published in a national or international journal of repute is essentially regarded as a substantiation of reliable and dependable studies carried out by a concerned research group. Manuscripts may be published in scientific journals as research articles, reviews, short communications, commentaries, proceedings, expert opinions or editorials. The most significant and popular types among them are ‘Research’ and ‘Review’ articles, which are frequently written, read, and popularized. After the author(s) put in a lot of effort and commitment to inscribe, each manuscript, when completed, is sent to a journal for publication, where it is selected depending on the topic of the manuscript and the broad field of the journal and its scope. Before sending the manuscript, it is the duty of the author(s) to understand the scope of the journal and make sure the topic of the manuscript fulfills the journals’ requirements. This will allow for avoiding unnecessary delays.

The Editor-in-Chief of the journal based on the potential of the topic and its suitability in the journal, decides in a meeting with the editorial staff whether the manuscript deserves to be sent for reviewing to the related reviewers. About 20-30% of the manuscripts can very quickly be categorized as unsuitable or beyond the scope of the journal. The Editor-in-Chief has the discretion to reject the manuscript straight off even before sending it to the reviewers for reviewing. Sometimes such a judgment can be made on the obvious quality of the manuscript; more often the quality may be high enough, but for one reason or the other it still does not fit the image and the scope of the journal. The editor(s) feel that the fairest treatment they can give authors is to notify them quickly of their decision. Thus, they reject the manuscript even without sending it to the reviewer or may send it only to one reviewer to confirm the decision.

Often after the process of reviewing, the most conspicuous, prominent, and outstanding article and its related manuscript will be the one winning the race among the other contemporaries, and the others may be declined giving reasons for rejection (reviewers’ comments). It seldom happens that a ‘stand out’ manuscript is accepted as such (without any improvements). Often for such articles the reviewer will ask the authors (through Editor-in-Chief) to improve upon the points listed to make it even more apposite and worthy of being published.

In this editorial the publication requirements of the two popular types of manuscripts, that is, research and review manuscripts, and the reasons for their denial have been discussed for fellow researchers, in particular for those who have entered the area recently, with the intention to share the prerequisite required for manuscripts and their submission to journals, including Journal of Young Pharmacists.

## RESEARCH MANUSCRIPT

A research article is a publication that illustrates one or more outcomes of a well-planned scientific research. A research manuscript is written by and for researchers, with the purpose of making specific findings known to the scientific community at large. Journals provide a protocol that is to be followed when writing a research article, in terms of the layout.

### Reasons for denial

There can be a number of reasons; the most prominent ones (non-limiting) are discussed:

#### Lack of Novelty, originality, and presentation of obsolete study

Novelty and unobviousness are the primary criteria that an editor of a scientific journal stresses upon the most. A mouth dissolving tablet preparation of a drug with conventional methods, technology, and / or known excipients presents no novelty to the existing state of the field unless the researcher demonstrates something new, adding to the existing knowledge. Also there is little or no scientific value in presenting an obsolete study, when newer methods are already available.

#### Improper rationale

The objective of doing research is to emphasize with proper justifications, backed by sufficient data. A controlled release formulation of a practically water insoluble drug may be denied on these grounds straight away. The whole manuscript should revolve along the rationale, which should be the central theme of the article. Usually the aim(s) and objective(s) should form the last sentence under the introduction section. Lack of focus and failure to adhere to the theme of the manuscript contributes to rejection. Probably in an attempt to have a voluminous article many manuscripts wander away from the objective, referring to things that are not within the scope of the study.

#### Unimportant and irrelevant subject matter

Publications in peer-reviewed journals are to disseminate knowledge. Therefore for a manuscript to be published in a well-recognized, international journal it must have significant scientific value. Again the editor is in search of something that is new and at the same time fulfills the requirements of the scope of his journal.

#### Flaws in methodology

Some manuscripts reflect improper methodology of the work done in the research study. This is attributed to the poor literature survey before starting the work, demonstrating the paltry knowledge of the researcher. A 300 mg tablet prepared using an 8 mm round punch will cause increased thickness in the tablet and is actually not suitable to be prepared. If this is shown in the manuscript it will make an unscientific impression in the mind of the reviewers. This may not be reflected in the reviewers’ comments to the author, but may be presented to the Editor-in-Chief in the confidential comments. If the methodology of a study is flawed or questionable, the result is bound to be flawed or questionable as well, and many highly rated peer-reviewed journals will not accept such a study.

#### Lack of interpretations

The researcher should have a sufficient know-how to interpret the exact reasons of the research outcome. Even if the results are out of specifications, the author should be able to critically interpret the cause in the discussion section. It is not mandatory to show positive outcomes alone. Manuscripts can support future research if they accurately interpret the root cause of the negative results.

#### Inappropriate or incomplete statistics

Application of statistics in the methodology and results sections of a manuscript creates an extra edge over the others, statistics being the need of the moment. Precisely showing the results with application of statistical principles will increase the probability of acceptance of the manuscript.

#### Reviewers’ field of knowledge and discretion

Sometimes, as an oversight the manuscript may be sent to a reviewer who may not be an expert in the field of the subject under review and he may give a casual glance to the manuscript deciding its eventual fate. In such cases it is believed that there are more chances of the manuscript getting accepted, however, the reverse may also happen. Although, in reputed international journals, the Editor-in-Chief will certainly consult another reviewer if the comments from one or two of them do not appear to be an outcome of critical evaluation.

#### Inappropriateness for the journal

The Editor-in-Chief always looks at the scope of the research study with respect to that of the journal before deciding whether to send it for reviewing. Some journal will look for research related to lead molecules rather than the existing and established drug molecules, unless the manuscript is out of the ordinary. Also the time of publication and the value of the particular subject matter being published in a journal are also critical.

#### Lack of in vivo studies

With the advent of sophisticated *in vivo* drug estimation technologies and methods to estimate the drug concentrations in minute quantities in a particular subject, a manuscript appears to be handicapped if the *in vitro* data is not supported by relevant *in vivo* findings and correlations. Acceptance of the manuscripts relying completely on the data generated solely through *in vitro* evaluations is something that is difficult in the present scenario.

#### Inappropriate packaging of the manuscript

In some cases, a less than borderline article may be published if well-packaged. In some cases an assessor finds it difficult to distinguish between ‘introduction’ and ‘discussion’. Introduction is to introduce the subject under research and to give the objective(s) and / or aim(s) of the article. The ‘discussion’ is to discuss the research, making references to similar studies done previously and interpreting the results obtained. ‘Materials and methods’ should be detailed enough so that any reader can duplicate the study. In fact this is good for verification of the authenticity of the study. The ‘Discussion’ should be relevant to the study. Previous studies that support or disagree with the present study should be mentioned. Impressions and guess work should be avoided. Any important statement that is not the direct result of the study should have a reference. The discussion should be limited to what has been studied.

#### Journals’ popularity and the priority given to the manuscript by the editor

Some manuscripts have potential, however, due to the popularity of the journal and due to the large number of hits to the journal the prospective manuscripts have to be declined as they face tough competition from the even superior research manuscripts kept in a higher grade by the Editor-in-Chief. However, if such a study is denied on these grounds, it sooner or later is able to find a fitting place in some other popular equally rated journal.

## REVIEW MANUSCRIPT

Review articles are an attempt to sum up the current state of the research on a particular topic. Ideally, the author(s) does an extensive literature survey and searches for *everything* relevant to the topic, and then sorts it all out into a coherent view of the ‘state-of-the-art,’ as it now stands. Review manuscripts describe the recent major advances and discoveries in a particular area of research, significant gaps in the research, and current debates and ideas of where research might go next.

Review articles are virtual gold mines if one wants to find out what the key articles are for a given topic. If one reads and thoroughly digests a good review article, he / she should be able to ‘talk the talk’ about that research topic. The key difference that distinguishes research from review articles is that the former strictly presents facts, rather than serving as a letter of opinion or a summary of the existing scientific literature. However, most scientific journals simultaneously publish such letters, as well as reviews of the body of existing research methods and findings.

### Reasons for denial

Major (non-limiting) causes for a review article being rejected have been discussed.

#### Lack of critical reviews, propaganda, and promotion of the techniques discussed

The most common comments received on evaluation of ‘ordinary’ review articles is the lack of critical observations and the opinion of the authors. Reviews are not just compilations; they are a mode of assessment of the previous researches done and are welcomed if the critical and judgmental opinions of the experts (authors) are incorporated. Simply collating the previous studies will not in any sense shape a review. Sometimes it appears as if the author is simply propagating and promoting the previous studies without his own personal outlook and critical reviews. The ‘grafting together’ of various statements from various authors without fully discussing the pros and cons of such statements will make the manuscript unacceptable for publication. A thorough understanding of the study followed by critical comments will create an edge rather than simply reproducing the text verbatim.

#### Inadequate and obsolete literature survey

A review article needs time, even when it is an invited review, and the Editor-in-Chief gives at least three to four months to the authors for critical and extensive literature survey as well as for the right compilation of such articles. More importantly, the literature survey done should include the most recent ones, as the reviews of the archaic studies might have already been published. Therefore, reproducing such published compilations will make lesser sense. An extensive literature survey done on the subject prior to writing a review will make a tentative representation or an illustration of the manuscript in the authors’ mind, which may take lesser time to cast into words.

#### Reviewer should be an expert on the subject

Similar to the case with research manuscripts, the reviewer needs to be an expert on the subject matter over and above the author(s), to critically analyze the authors’ opinions on the subject. Preferably the reviewer selected for reviewing should have prior or present research experience in the subject, to be able to assess the manuscript meticulously.

#### Editor-in-chief looking for something specific at a particular time

Sometimes a particular research area is emphasized upon at a particular time, based on its present need and importance as also on its usefulness and potential in the future or theme issue. Considering today’s scenario, for example, if an author is working on a review article that encompasses all the recent delivery systems for effective treatment of swine flu, it will be of palpable interest to publish the manuscript. Obviously the editor will go for a peer review (and may reject it, if found unsuitable); the only thing that is to be emphasized is the fact that this provides priority to the manuscript for peer review and publication.

The reasons discussed earlier are chiefly the ones that are related to the manuscript type (research / review), due to which the manuscript becomes liable for rejection. Other additional reasons that may play their part adding to the above-mentioned reasons, irrespective of the journal type may be:

- Favoritism or partiality based on the country of origin of the manuscript (less often, but still cannot be completely ruled out)
- Missing Conflict of Interest statement
- Improper manuscript uploading in the journals’ author center (this may add to the frustration of the Editor-in-Chief)
- Missing covering letter or with improper authors’ affiliations
- Improper formatting and language, grammatical lapses, and typographic errors
- Inadequate corrections of galley proofs: Galley proofs should be corrected ‘boldly’ preferably with a red pen so that the printer can easily see it, and not corrected on a separate sheet of paper. In addition most journals send them along with author queries (AQ) and instructions to be followed for correcting the galley proofs
- Inappropriate reference citations ignoring the journals’ format
- Abstract not given as per journals requirements: Some journals require a brief (< 200 words) synopsis clearly outlining the scene and article scope, briefly putting it into context. The aim of the abstract is to draw in the interested reader, so a clearer and more insightful abstract will generate more interest and will make the manuscript attractive. In most of the journals, the following structure is advised nowadays to make the most of the abstract: *Background*: Provides a brief defining statement about the area under discussion and its importance *Objective*: What questions did you set out to address? *Methods*: How did you define the scope of the manuscript / what were the limits of your literature search? *Results / Conclusion*: What were the most important findings overall?
- Other factors such as file formatting, spacing, and headings, units and abbreviations, spellings, companies and drug brand names, bibliography, tables, illustrations, chemical structures, submission deadlines (in case of invited reviews), copyright forms submission at appropriate time, grammatical and syntax errors, and so on are all to be taken care of for every specific journal, having distinct requirements for each. Failure to meet the above-mentioned criteria may not necessarily reject the manuscript, but can delay the publication of your manuscript adding to unnecessary afflictions.

## MANUSCRIPT REJECTION: IS IT THE END OF THE WORLD FOR THAT MANUSCRIPT?

Obviously not. Realize there are many reasons for rejection. As stated earlier, publishers are often looking for something quite specific. Do not be disappointed and do not take it personally. An unknown person reads your manuscript and makes a judgment based on certain well-established criteria. Respect his comments, one should be mature enough to understand the comments whether they are genuinely written or have been written just for the sake of writing. Invigorate yourself, read your article again, asking yourself if it could be improved in any way, along with the comments of any of the reviewers. Authors have to become their own best editor, best critic, and strongest supporter. Read your article with a questioning mind to see if it strolls off the subject at some point, or states a problem which it does not solve, or repeats the same point several times. Ask your competitor or peer group having the knowledge of the subject matter to read it giving additional comments. One may rewrite a part or may improve it by doing some more research to try and find a better match. Based on reviewers’ comments, improve upon the manuscript and send it to the same journal again. If one journal rejects your article, try another one, and another one, and yet another one!

In summation it is true that performing research is not a walk in the park, to add to the obscurity, getting your manuscript to be accepted and published in a reputed journal makes the matter even more convoluted. It will actually take you to the field of competition. Reputed journals by no means face scarcity, with potential manuscripts queued up to get published, but high-degree research and corresponding well-drafted manuscripts are the ones that will eventually win the race. Emphasize more on the quality of research rather than being into the numbers game: just to fill the pages of the curriculum vitae! Many highly reputed journals are not likely to accept such articles. The key: Give yourself as well as the manuscripts the time they deserve and come up with a luminary manuscript. Keep thinking, give some time daily and you will be served with new ideas / thoughts / phrases / texts to improve upon your manuscript taking it to the extremes of betterment.

Wishing the readers happy researching for their scientific writing.

